# Different memory T cell phenotypes are elicited by Ad5 and rare adenoviruses

**DOI:** 10.1186/1742-4690-9-S2-P246

**Published:** 2012-09-13

**Authors:** P Penaloza, E Borducchi, A McNally, N Simmons, J Teigler, N Provine, W Tan, R Ahmed, DH Barouch

**Affiliations:** 1 Beth Israel Deaconess Medical Center, Boston, MA, USA; 2 Emory University, Atlanta, GA, USA

## Background

The anamnestic potential of memory T cells are pivotal components of the adaptive immune response. Analyzing memory T cells elicited by different vaccine vectors is crucial for the identification of novel vaccine modalities against HIV.

## Methods

6-8 week old C57BL6 mice were used for these experiments. Mice were immunized intramuscularly with 10^10 VP in PBS. The different adenoviral vaccines (Ad5, Ad26, Ad35, Ad48) expressed the glycoprotein (GP) derived from lymphocytic choriomeningitis virus (LCMV). For challenge studies, the mice were infected with 2x10^6 PFU of LCMV Cl-13 intravenously. Early and late CD4 and CD8 T cell recall was measured by flow cytometry. Viral control was assessed by standard plaque assay on VERO cells.

## Results

We have compared T cell memory phenotypes and immune protection elicited by common adenoviruses serotype 5 (Ad5) versus rare adenoviruses serotypes (Ad26, Ad35, and Ad48). For these comparative studies, we immunized mice with non-replicating adenovirus vectors expressing the lymphocytic choriomeningitis virus (LCMV) GP, and challenged with chronic LCMV Cl-13. Our comparative data show that Ad5-GP generates an increased magnitude of LCMV GP-specific CD8 T cell responses (compared to that generated by rare adenoviruses). However, GP-specific CD8 T cells elicited by Ad5-GP immunization express inhibitory PD-1, and produce reduced amounts of cytokines, suggesting qualitative defects in memory CD8 T cells. This unexpected expression of PD-1 may also reflect antigen-persistence of Ad5.

## Conclusion

Memory T cells elicited by rare adenovirus-based vaccines results in greater T cell recall potential after viral challenge, and higher functionality compared to vaccination with Ad5. These data suggest that alternative serotype Ad vectors may offer substantial benefits over Ad5 as vaccine vectors in eliciting optimal T cell memory to chronic viruses such as HIV, HBV or HCV.

